# Long-term influence of applied dentin sealing techniques and luting material type on the bond durability of a resin CAD/CAM restorative material

**DOI:** 10.1186/s12903-026-08682-z

**Published:** 2026-06-01

**Authors:** Ghadeer Abo-Eldahab, Mohamed Kamel, Omaima Ghallab

**Affiliations:** 1 Department of Operative Dentistry, Faculty of Dentistry, Ainshams University, Cairo, Egypt; 2 Faculty of Dentistry, Ainshams University, Organization of African Unity St, El-Qobba Bridge, El Weili, Cairo, Cairo Governorate Egypt

**Keywords:** Immediate dentin sealing, CAD/CAM, Bond strength, Self-adhesive resin cement, Flowable composite, Bond durability

## Abstract

**Background:**

The durability of bonded CAD/CAM restorations depends largely on effective adhesion to dentin. Immediate dentin sealing (IDS) has been proposed to enhance bonding effectiveness compared with conventional delayed dentin sealing (DDS). Additionally, while resin cements remain the gold standard for luting, flowable composites have emerged as potential alternative. This study aimed to evaluate the effect of different dentin sealing techniques and luting materials on the microtensile bond strength (µTBS) of CAD/CAM composite disc to dentin after immediate and long-term aging.

**Methods:**

Twenty flat dentin specimens were obtained from extracted human molars and randomly assigned to two main groups according to the dentin sealing techniques into (group 1: No-IDS and group 2: IDS with a universal adhesive). The two groups were provisionally restored with temporary materials for two weeks. Afterwards the temporary materials were removed, and surface was reactivated with air borne abrasion. Each group was subdivided according to the luting material used for CAD/CAM composite discs into (subgroup 1: dual-cure self-adhesive resin cement and subgroup 2: submicron hybrid flowable composite). The specimens were sectioned into micro-bars and tested for µTBS either after 48 h (immediate) or 6 months (long-term aging) in water storage. Data were analyzed using three-way ANOVA and post-hoc comparisons (α = 0.05). Failure modes were analyzed.

**Results:**

Immediate µTBS values displayed no significant differences among the tested groups. Following long-term aging, IDS combined with a flowable composite yielded significantly higher µTBS compared to groups without IDS. Furthermore, the flowable composite demonstrated significantly higher bond strength than the self-adhesive resin cement, regardless of the dentin sealing protocol used.

**Conclusions:**

The microtensile bond strength of CAD/CAM composite to dentin is dependent on both the dentin sealing technique and the luting material. Immediate dentin sealing combined with a submicron hybrid flowable composite demonstrated the highest bond strength after long-term aging, compared to the tested self-adhesive resin cement. The effect of artificial aging was highly material-dependent; therefore, long-term aging is necessary to accurately evaluate the bond durability.

## Background

The extensive damage of tooth structure due to caries, fracture or wear may favour selection of definitive indirect restoration over direct solution [1]. Various computer-aided design and computer-aided manufacturing (CAD/CAM) materials are currently available for fabrication of dental restorations with high esthetics while preserving optimal mechanical properties [2]. CAD/CAM resin composite have been introduced to overcome the brittleness of traditional ceramics and the sophisticated direct application technique of composite restoration in addition to the advantage of easy precise milling, high mechanical performance and intraoral repairability [3]. However, long-term survival of non-retentive indirect restorations does not rely exclusively on the restorative material used. It mainly depends on efficient adhesion and luting cementation between the restoration and the prepared tooth structure [2, 4]. However, extensive loss of tooth structure causes exposure of substantial amount of dentin. Success of adhesion to dentin is less predictable than enamel due to intrinsic complex hydrophilic nature of dentin [5]. Hence, durable aging-resistant adhesion to dentin is critical for clinical survival of partial indirect restorations [6].

In the 1990s, Paul et al. and Bertschinger et al. introduced the concept of “dual bonding” technique which prescribes an immediate application of adhesive after cavity preparation. While “resin coating” technique recommends additional application of a low-viscosity resin composite over the adhesive [7]. The purpose of these techniques is to minimize contamination during provisional cementation and enhance bond strength. Later Magne et al. proposed the immediate dentin sealing (IDS) concept that shares a similar concept with the proposed resin coating immediately for sealing freshly cut dentin [8]. Furthermore, IDS recommended the use of filled bonding agent as an alternative to the use of unfilled adhesive followed by low-viscosity resin composite.

Several studies, clinical trials and meta-analyses evaluated the benefits of IDS over the traditional delayed dentin sealing (DDS) which involves applying the adhesive later prior to restoration cementation. These highlighted the possible advantages of reducing dentin permeability, postoperative sensitivity, contamination, as well as enhancing bond strength and resistance of hybrid layer to polymerization shrinkage stress during final cementation [9–12]. Regardless of the vast research, the impact of IDS on the performance of partial indirect restorations remains questionable. Recent systematic reviews and meta-analyses reveal lack of appropriate long-term studies justifying the benefits of IDS over DDS [13–18]. Moreover, recent evidence presented non-significant difference between IDS and DDS prior to indirect restorations confirming conflict findings [19–21].

While the three-step etch and rinse adhesive is considered the gold standard between other adhesive systems, there is a trend of simpler less complicated techniques. Moreover, the challenge of achieving durable stable adhesion to dentin has led to continuous innovations in dental adhesive technology reaching the universal adhesives [22, 23]. The “universal”, or “multimode” adhesives can be used in several bonding strategies either etch-and-rinse, self-etch, or selective enamel-etch mode. Universal adhesives have been advanced enough to form reliable bond between tooth substrates and restorative materials, with outstanding medium-term longevity [24]. As universal adhesives are basically considered a type of one-step self-etch adhesives, the durability and stability of their bonding interface remain questionable [25].

The emergence of dual-cure self-adhesive resin cements marked a continuation of the simplification trend for indirect restorations cementation [26]. Further compositional enhancement introduced adhesive/ self-adhesive resin cement systems to be used separately or combined universally according to clinical preferences [24]. These modifications allow the universal cement and adhesive to work synergistically without incompatibility concerns with optimal adhesion to both dental tissues and versatile restorative materials. Among commercially available products, SoloCem (Coltène/Whaledent, Switzerland), in conjunction with OneCoat 7 Universal, is marketed as a universal cement-adhesive system. These materials incorporate functional acidic monomers—notably 10-methacryloyloxydecyl dihydrogen phosphate (10-MDP)—which play a critical role in chemical bonding to dentin and enamel. The 10-MDP molecule forms stable calcium salts with hydroxyapatite and hydrogen bonds with collagen, contributing to a durable adhesive interface. In addition, 10-MDP and its calcium complexes have been shown to inhibit matrix metalloproteinases (MMPs), thereby limiting collagen degradation and promoting hybrid layer longevity [27]. Despite SoloCem’s classification as a universal cement, there remains a notable absence of independent data evaluating its bonding effectiveness to tooth structures or CAD/CAM restorative materials. This lack of evidence emphasizes the need for further investigation to substantiate its clinical claims and validate its performance in comparison to light- cured flowable resin composite.

While resin cements are considered the gold standard for adhesive cementation with accepted mechanical properties, attention has diverted to flowable resin composite as a valuable alternative to resin cements [2]. Advanced adhesive techniques combined with recent flowable formulations through optimized filler system have demonstrated the potential to narrow the performance gap present in resin cements [28]. The viscosity of flowable composites allows easy removal of excess material without the need for partial or tack curing, as is often required with resin cements. Their low viscosity also enables thin film thickness and accurate seating of restorations under minimal pressure. While their single-component nature lowers the risk of void entrapment compared to dual-component resin cements [2].

Therefore, this study aimed to evaluate the influence of different dentin sealing techniques and luting material types on the microtensile bond strength (µTBS) of CAD/CAM composite discs to dentin under immediate and long-term aging. Therefore, the null hypotheses tested were that the microtensile bond strength of CAD/CAM discs to dentin would not be significantly affected by (1) the dentin sealing technique, (2) the type of luting material, or (3) the aging period.

## Materials and methods

### Sample size calculation

Power analysis was designed to have a significant difference between the tested groups regarding micro-tensile bond strength. By adopting an alpha (α) level of 0.05 (5%), a beta (β) level of (0.2) (i.e. power = 80%) and an effect size (f) of (0.42) calculated based on the results of a previous study.^1^; the predicted total sample size was found to be (72) samples (*n* = 6). Sample size calculation was performed using G*Power version 3.1.9.7^2^. However, the sample size was increased to 20 to ensure more accuracy.

### Study design

**Fig. 1:**
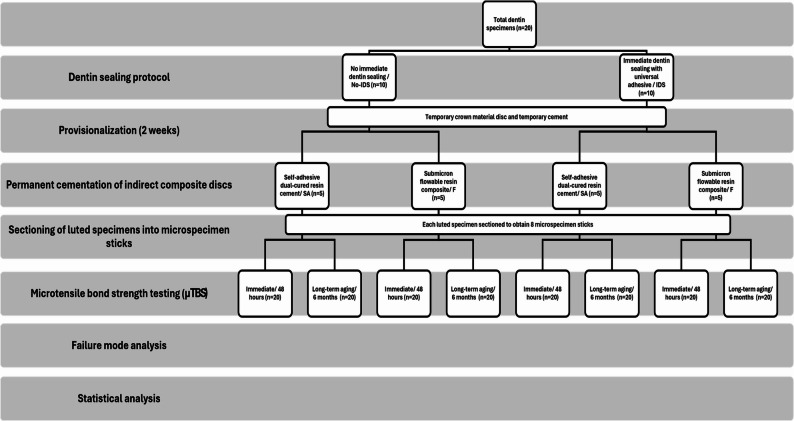
Schematic illustration of the experimental workflow

A total of 20 dentin specimens were prepared and randomly divided according to the dentin sealing technique into two main groups; group 1: No immediate dentin sealing / No-IDS and group 2: Immediate dentin sealing with universal adhesive / IDS (*n* = 10). The dentin specimens were provisionally restored with temporary crown material discs and temporary cement. After 2 weeks, the temporary discs and cement were removed, and specimens of each main group were randomly divided into two subgroups according to the resin-based materials used for permanent cementation of the overlying CAD/CAM composite discs; subgroup 1: self-adhesive dual-cured resin cement/ SA and subgroup 2: submicron flowable resin composite/ F) (*n* = 5). After luting, each luted specimen in the subgroup was vertically sectioned to obtain 8 equal ~ 0.9 mm^2^ bars, which were equally divided and randomly allocated into two subdivsions according to the timing of testing the µTBS; subdivision 1: after 48 h / immediate and subdivision 2: after 6 months in distilled water storage / long-term aging; a total of 20 bars in each subdivision collected from 5 luted specimens (*n* = 20). The workflow of the study is shown in Fig. 1. The details of the materials composition and manufacturer are detailed in Table 1 for the materials used in this study.

**Table 1:** The details of the materials composition and manufacturer

Materials	Description	Composition	Manufacturer	Lot number
One coat 7 universal	Universal light-curing single-component bonding agent	HEMA, MMA-modified polyacrylic acid, UDMA, amorphous silicic, 10-MDP, ethanol, water, Water, ethanol 40–50 wt% Methacrylate monomers 25–35 wt% 10-MDP, polyacrylic acid 10–20 wt% Fillers (amorphous silica, SiO2) 1–10 wt% Ph = 2.8	Coltène, Whaledent, AG, Switzerland	M31361
Brilliant EverGlow Flow A2 / B2	Submicron hybrid flowable resin composite	Bis-GMA, BisEMA, barium glass, silanized amorphous hydrophobic silica Filler content: 65 wt%, 46 vol%		M33939
Solocem (Translucent)	Self-adhesive dual-cured resin cement	2,6-di-tert-butyl- 4-methylphenol, 10-MDP, dibenzoyl peroxide (BPO initiator), 4-META, TEGDMA, DUDMA, Bis-GMA, HEMA, zinc oxide, Ytterbium (III) fluoride		M33732
BRILLIANT Crios (A2 14)	Nano-hybrid composite blocks	Cross-linked copolymer of methacrylate derivatives (TEGDMA, Bis-EMA, UDMA, Bis-GMA) 23–35 wt%, Barium aluminum boron silicate glass (< 1.0 μm) 50–76 wt%, Amorphous silica (SiO2) (˂20 nm) 1–10 wt%, inorganic pigments such as ferrous oxide or titanium dioxide		M43668
TempoSIL 2 (white)	Temporary cement	Zinc oxide Polydimethylsiloxane		M40144
Cool Temp Natural (A2)	Two-component short-term temporary crown and bridge material	Methacrylates, Barium glass silanized, Amorphous silica hydrophobed		M24796

*10-MDP* 10-Methacryloyloxydecyl dihydrogen phosphate, *UDMA* Urethane Dimethacrylate, *Bis-GMA* Bisphenol A diglycidylmethacrylate, *DUDMA* Diurethane dimethacrylate, *4-META* 4-methacryloxyethyl trimellitic anhydride, *HEMA* 2-Hydroxymethacrylate, *TEGDMA* Triethyleneglycol dimethacrylates;, *SiO2* Silicone dioxide

### Preparation of dentin specimens

A total of 20 freshly extracted, sound, non-carious and non-restored human molars were collected from patients between the ages of 45–60 years visiting the Oral Surgery Clinic of Ain Shams University for periodontal purposes. This study was carried out according to the guidelines and regulations of the Ethical Committee of Faculty of Dentistry, Ain Shams University. The teeth were cleaned under running water, and all soft tissues and hard deposits were removed using hand scaler (Martin GmbH, Germany). The collected teeth were stored in 0.5% Chloramine-T at 4°c and used at maximum 1-month post extraction.

Custom- made circular polyurethane mould, with 2 cm thickness and 20 cm in diameter was fabricated, with five circular holes of 2 cm depth and 2 cm diameter. Separating medium was applied to the walls of each hole then fast-set self-cured acrylic resin (Dots acrylic, Egypt) was poured inside each hole. Molar roots were embedded into the dough stage of the acrylic resin, 2 mm below the cementoenamel junction (CEJ). After complete setting, each specimen was removed from the mould and the occlusal coronal one third of the crown was removed under copious air-water coolant spray with diamond disc (Honeycomb Design 6924, KOMET, USA). The dentin surface was abraded with #600 grit wet silicon carbide paper for 30 s using a circular motion to create a standardized flat surface and clinically simulated smear layer [29, 30]. The specimens were ultrasonically cleaned in distilled water for 2 min to remove any remaining debris. The dentin surfaces were examined using a stereomicroscope to verify the absence of remaining enamel.

### Fabrication of CAD/CAM resin composite disc specimens

Seven CAD/CAM resin composite blocks (BRILLIANT Crios, Coltene, Switzerland) were used to fabricate the indirect composite disc specimens. Size-14 blocks were used, measuring 14 mm in width, 12 mm in length, and 18 mm in height. Each block was sectioned horizontally using a slow-speed diamond disc under copious water cooling (IsoMet 4000, Buehler, Lake Bluff, IL, USA). This process yielded three resin composite discs per block. The final discs had a uniform thickness of 3 mm, maintaining the original width (14 mm) and length (12 mm).

### Dentin sealing techniques

The dentin specimens were randomly divided into two main groups (*n* = 10) depending on the dentin sealing technique.

- Regarding the No-IDS group, the dentin was not sealed immediately after preparation.
- For the IDS group, One Coat 7.0 Universal adhesive was used to seal the dentin surface immediately after preparation. The adhesive bottle was well shaken each time before use in this study. A drop of the adhesive was dispensed into a mixing well and rubbed onto the dentin surface for 20 s using a disposable microbrush, then gently dried with oil-free compressed air for 5 s. The tip of light-emitting diode (LED) (3 M Espe Elipar, Germany) was applied perpendicular to the dentin surface, and the adhesive was light cured for 10 s. Additional light curing was done for 10 s through a layer of glycerin gel as a final step of curing, to ensure curing of the oxygen inhibited layer. Glycerin was thoroughly rinsed with air/water spray for 10 s and air dried for 10 s. The output intensity of LED light curing unit was measured regularly after each group using light intensity meter to ensure that the value is ≥ 1400 mW/cm^2^ tthroughout the study.

### Fabrication of temporary discs and temporary cementation

Circular transparent silicone mould (15 mm inner diameter x 2 mm thickness) was used to fabricate temporary restoration disc. The temporary material (Cool Temp Natural, Coltene, Switzerland) was dispensed with the mixing tip into the mould to set against two microscopic glass slides. The disc was removed after 60 s and left for 270 s to ensure complete setting. Temporary cement (TempoSIL 2, Coltene, Switzerland) was applied through a mixing tip to cement the temporary disc over the dentin surface with slight pressure undisturbed for 60 s. After setting for 2 min, the excess cement was removed and each specimen was stored in separate labeled container in distilled water for two weeks in an incubator (BTC, Model: BT1020, Cairo, Egypt) at 37 °C, and the water was replaced every three days to prevent bacterial and fungal growth.

### Permanent cementation of dentin specimens with CAD/CAM resin composite discs

After two weeks of provisional restoration, the temporary discs and cement were carefully removed with excavator (Maillefer Excavator 51/52, Dentsply Switzerland) to avoid scratching the surface. In addition, the dentin surface was cleaned by air abrasion (Aries Outlets, China) with 50 μm aluminium oxide particles at 10 mm distance following a 45º angulation for 5 s at 2 bar pressure. Dentin surface was subsequently rinsed thoroughly with water for 20 s and air-dried for 10 s.

**Fig. 2:**
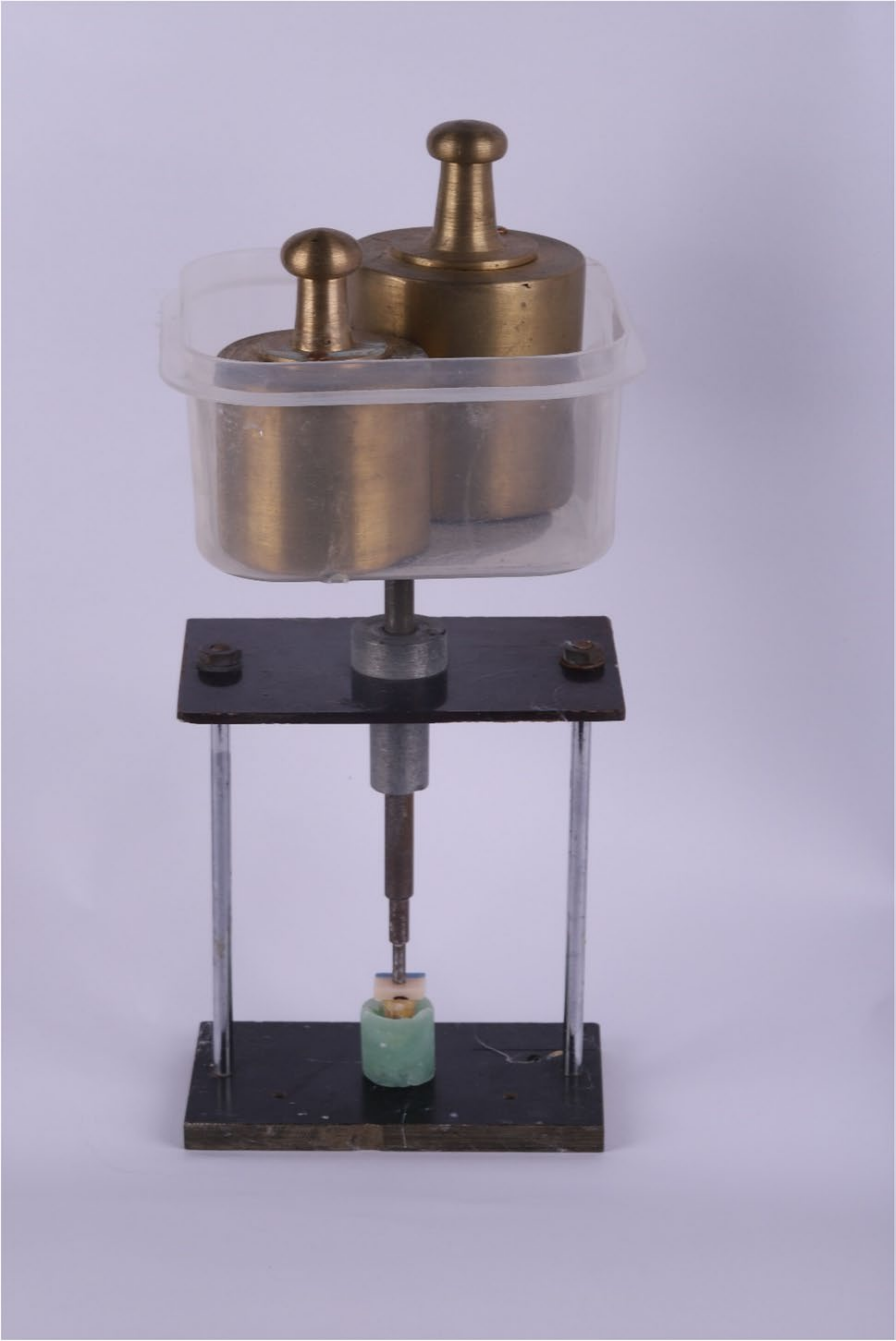
Loading device applying constant pressure (3KG) over the dentin specimen during cementation with overlying CAD/CAM resin composite disc

Immediately before cementation, the planned bonding surface of indirect composite disc were sandblasted using 50 μm Al_2_O_3_ at 10 mm for 10 s with 1.5 bar according to manufacturer instructions. The sandblasted surface was ultrasonically cleaned (Eumax^®^, Hong Kong Model number: UD80SH-2.6 L) in distilled water for 5 min and air-dried for 10 s. A drop of OneCoat 7 Universal adhesive was applied on the sandblasted surface, rubbed with disposable dental brush for 20 s, gently dried with oil-free compressed air for 5 s and left uncured covered with black cover mixing well. Each resin composite disc was cemented on the dentin specimen with the corresponding resin-based luting material:

- Subgroup 1: Self-adhesive dual-curing resin-based luting cement (SoloCem, Coltene, Switzerland) was used as luting material without prior application of adhesive. A small amount of the cement was extruded and wiped to ensure optimum mix, then homogenous paste was applied using the auto-mix tip supplied by the manufacturer over the composite disc surface. The resin composite disc specimen was placed over the refreshed dentin surface and seated by light pressure. A constant pressure of 3 kg (29.41995 N) was immediately placed over the luted assembly by means of a metal tool for complete seating of the indirect composite disc as in Fig 2. The excess resin cement was carefully removed with a microbrush. The seating load was applied for the first 2 min allowing the dual-cured resin cement to set in primarily self-curing mode (in ambient room light). Subsequently, the resin cement was photopolymerized from the four sides of the top surface of the luted specimen each for 40 s. Additional photopolymerization was done for the other four lateral directions (mesial, distal, buccal, and lingual) with light-curing tip as close as possible to the interface each for 40 s. The seating load was left in position for 10 min then the luted specimen was removed out of the loading device. All bonding procedures were conducted at room temperature (23 ± 1 °C) and at 60% relative humidity.
- Subgroup 2: Universal adhesive (One Coat 7.0 Universal, Coltene, Switzerland) and submicron hybrid flowable composite (Brilliant EverGlow Flow, Coltene, Switzerland) group were used as luting material. A drop of the adhesive was dispensed in a mixing well and applied on the dentin surface with a disposable microbrush for 20 s, then gently air-dried for 5 s and light cured for 10 s. The flowable composite was applied with the applicator tip over the resin composite disc specimen. The resin composite disc specimen was placed and seated over the dentin surface by light finger pressure. The same seating load as in subgroup 1 was used to hold the resin composite disc specimen in place while the flowable composite was photopolymerized from the four sides of the top surface of the luted specimen each for 40 s. Additional photopolymerization was done for the other four lateral directions (mesial, distal, buccal, and lingual) with light-curing tip as close as possible to the interface each for 40 s, then the luted specimen was removed out of loading device.

Each luted specimen was stored in 5 ml distilled water in separate sealed container labelled with group name and specimen number in an incubator (BTC, Model: BT1020, Cairo, Egypt) at 37 °C for 48 h before sectioning.

### Preparation of microtensile specimen sticks

Subsequently, the luted specimens were sectioned to obtain microspecimen sticks for immediate and delayed µTBS testing. Sectioning was done perpendicular to the interface using a water-cooled linear precision diamond saw (IsoMet 4000 Buehler, Lake Bluff, IL, USA), to obtain rectangular microspecimen sticks per molar (~ 1 × 1 × 6–7 mm). A total of eight microspecimen sticks were collected from the middle part of each sectioned specimen with dentin thickness ≥2 mm, whereas four of them were randomly selected for immediate µTBS testing and the other four microspecimen sticks were assigned for the long-term aging µTBS testing. A total of 20 microspecimens (collected from 5 luted specimens) were assigned per experimental subdivision according to the timing of testing the µTBS (*n* = 20).

### Microtensile bond strength testing

For immediate µTBS testing, the microspecimen sticks were tested immediately after sectioning. For the long-term µTBS testing, each microspecimen stick was stored separately in 10 ml distilled water in sealed container labelled with the name of experimental group, the number of luted specimens collected from and the number of the stick, in an incubator (BTC, Model: BT1020, Cairo, Egypt) set at 37 °C for 6 months before testing. The distilled water was replaced weekly to prevent contamination.

**Fig. 3:**
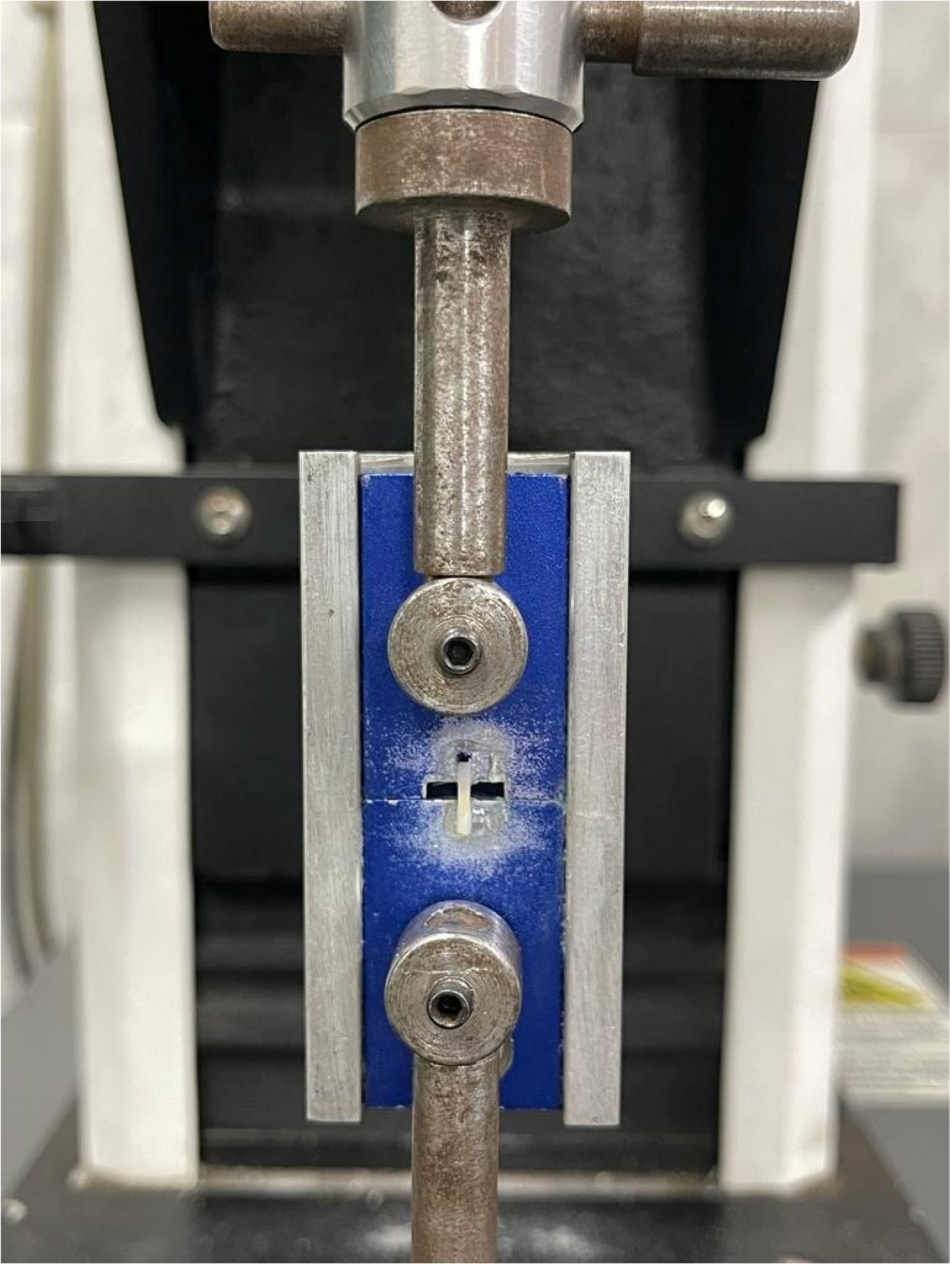
Microspecimen stick was fixed with cyanoacrylate glue on the µTBS universal testing machine

The µTBS test was carried out using a universal testing machine (Instron 3345, Instron Corporation, USA). The microspecimens were fixed using cyanocrylate glue (Model Repair II Blue, Dentsply-Sankin, Tokyo, Japan), away from the adhesive interface, to a modified µTBS testing jig as in Fig. 3. The microspecimen was tested in tension mode at a crosshead speed of 1.0 mm/min with a load cell of 100 N until failure. The dimensions of the sticks were precisely measured with digital calliper (CD-15CPX, Mitutoyo, Kanagawa, Japan) from which the cross-sectional area was estimated. The mean value of µTBS was calculated (in MPa) with a computer software (BlueHil universal Instron, England) through dividing the employed force (in N) at the time of fracture by the measured bonded area (in mm^2^). Specimens that failed before actual testing (pretesting failure) were noted and not taken into interpretation and calculation of the µTBS means.

### Failure mode analysis

After the debonding, the failure patterns were assessed using a stereomicroscope (Wild M5A, Wild) at a magnification of 50x and according to the overall failure mode of the micro-specimen it was classified as: cohesive in dentin, adhesive at the luting material-dentin interface, cohesive in the adhesive luting material, adhesive at the luting material- indirect composite interface, hybrid involving mixed interfacial failure and cohesive in the indirect composite; guided by the Academy of Dental Materials [29, 31].

### Statistical analysis

Numerical data were explored for normality by checking the data distribution, calculating the mean and median values and using Kolmogorov-Smirnov and Shapiro-Wilk tests. Data showed parametric distribution so; it was represented by mean and standard deviation ^***(SD)***^ values. Three-way ANOVA was used to study the effect of different tested variables and their interaction. Comparison of main and simple effects were done utilizing pairwise t-tests with Bonferroni correction. The Comparison between groups with qualitative data was done by using Chi-square test. The significance level was set at *p* ≤ 0.05 within all tests. Statistical analysis was performed with IBM^®^ SPSS^®^ Statistics Version 26 for Windows.

## Results

### Immediate and long-term microtensile bond strength (µTBS) of indirect resin composite specimens to dentin sealed using different techniques and cemented with different luting materials

The three-way ANOVA revealed that both the dentin sealing technique (*p* = 0.024) and the luting material types (*p* = 0.001) had a statistically significant main effect on bond strength. However, aging alone showed no statistically significant difference as a main effect (*p* = 0.428). Regarding interactions, a statistically significant difference was found for the two-way interaction between the luting materials and aging (*p* = 0.022), indicating that the influence of aging was highly dependent on the type of luting material used. All other two-way interactions, as well as the three-way interaction between dentin sealing, luting material, and aging, showed no statistically significant difference (*p* > 0.05).

Table 2 presents a comparative analysis of mean and standard deviation values of immediate (48 h) and long-term aging (6 months) µTBS based on the type of luting material (SA vs. F) and the dentin sealing technique (IDS vs. No-IDS). The highest statistically significant difference mean µTBS was recorded in the IDS-F group after aging (27.71 ± 8.00 MPa), compared to all other tested groups.

**Table 2:** A comparative analysis of mean and standard deviation values of immediate (48 h) and long-term aging (6 months) µTBS based on the type of luting material (SA vs. F) and the dentin sealing technique (IDS vs. No-IDS)

Dentin sealing technique	Luting materials	Aging	
		Immediate (48 h)	Long-term aging (6 months)
No-IDS	SA	15.70 ± 3.35Aa	13.41 ± 3.87Ac
	F	18.36 ± 5.39Aa	21.30 ± 5.69Ab
IDS	SA	18.92 ± 4.60Aa	16.23 ± 3.22Abc
	F	20.68 ± 3.23Ba	27.71 ± 8.00Aa

Using: Three-way analysis of variance

Different Uppercase letters indicate significant differences in the same row (*p* < 0.05). Different Lowercase letters indicate significant differences between groups in the same column (*p* < 0.05)

Immediate µTBS results display statistically non-significant difference between all immediate tested groups. However, after aging, the IDS-F group yielded a higher statistically significant µTBS than the No-IDS-F group (27.71 ± 8.00 MPa vs. 21.30 ± 5.69 MPa). Also, after aging, the F groups demonstrated higher statistically significant in the µTBS than the SA groups within the No-IDS technique (21.30 ± 5.69 MPa vs. 13.41 ± 3.87 MPa) as well as within the IDS technique (27.71 ± 8.00 MPa vs. 16.23 ± 3.22 MPa). Finally, there was no statistically significant difference between the immediate and aged µTBS for all groups except with the IDS-F groups, aging resulted in a statistically significant higher µTBS from 20.68 ± 3.23 MPa to 27.71 ± 8.00 MPa.

### Failure mode analysis

**Fig. 4:**
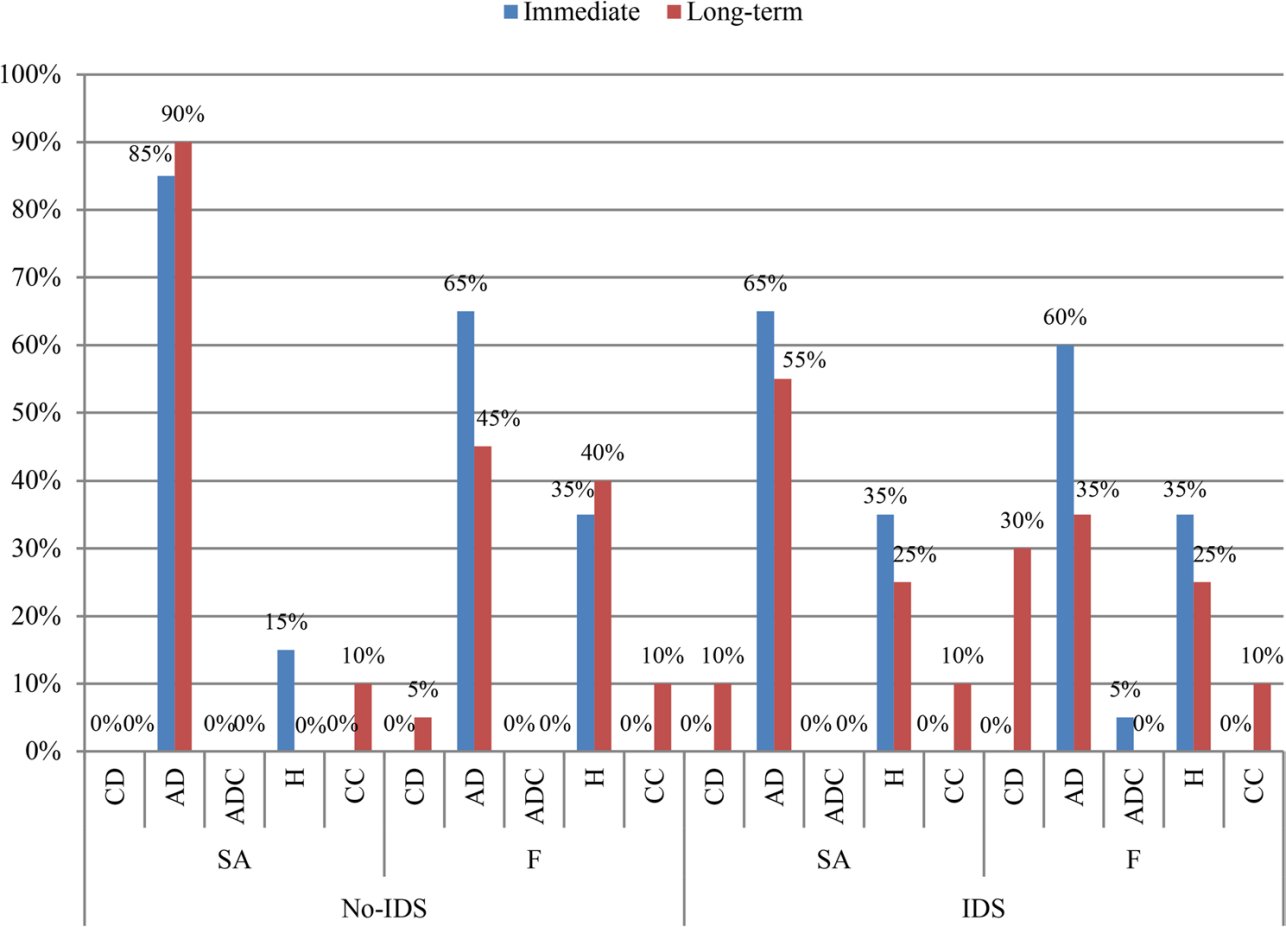
Bar chart displays failure mode analysis of Specimens treated with different dentin sealing techniquees (IDS vs. No-IDS) and luting materials (SA vs. F) within tested aging periods (Immediate vs. Long-Term). *CD: Cohesive failure in dentin; AD: Failure at interface dentin/cement; ADC: Failure at interface cement/indirect composite; H: Hybrid failure at both interfaces; CC: Cohesive failure in composite*

**Fig. 5:**
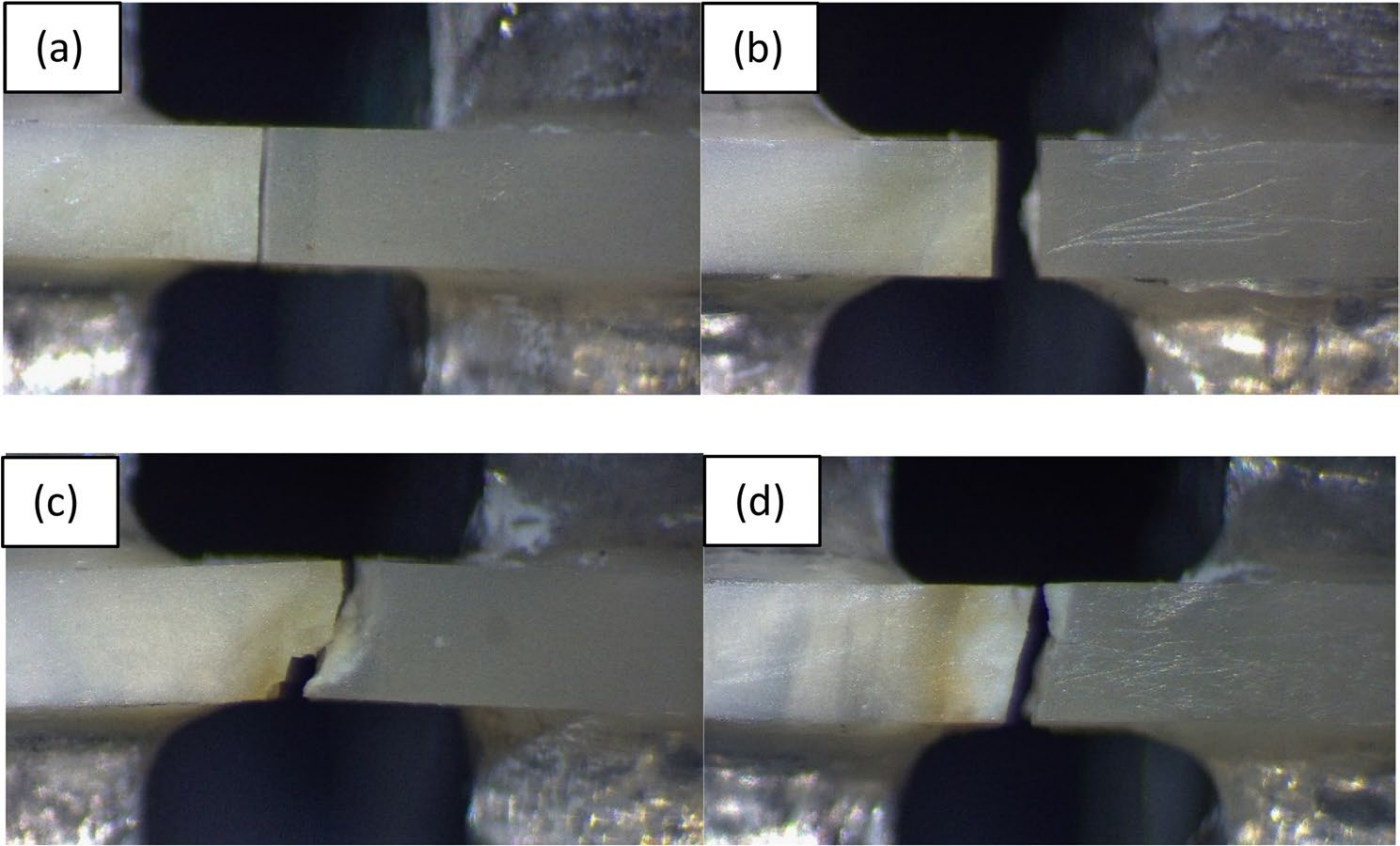
Failure mode patterns. **a** Adhesive failure at dentin/cement interface (AD); **b** Cohesive failure in the adhesive luting material (CC); **c** Cohesive failure in the dentin (CD); **d** Hybrid failure at both interfaces (H)

Failure mode analysis of specimens treated with different dentin sealing techniques (IDS vs. No-IDS) and luting materials (SA vs. F) within tested aging periods (Immediate vs. Long-Term) are represented in Table 3 and Fig. 5. Stereo microscope images of different failure mode analysis of representative samples are shown in Fig. 4. Adhesive failure at the dentin–cement interface (AD) was the most frequent failure mode in all groups. A statistically significant difference (*p* = 0.031) in failure mode distribution was observed within the IDS-F groups when comparing immediate and aged specimens. Specifically, long-term aging induced a notable transition from predominantly adhesive failures (AD decreased from 60.0% at the immediate phase to 35.0% post-aging) to an increased incidence of cohesive dentin failures (CD increased from 0.0% to 30.0%). Additionally, while the distribution of failure modes among the different dentin sealing and luting materials showed non statistically significant difference at the immediate testing baseline (*p* = 0.415), a statistically significant difference emerged across the groups following long-term aging (*p* = 0.007). There was no pretest failure observed before testing.

**Table 3:** Failure mode analysis of Specimens treated with different dentin sealing techniques (IDS vs. No-IDS) and luting materials (SA vs. F) within tested aging periods (Immediate vs. Long-Term)

Dentin sealing technique	Luting materials	Failure mode	Aging				*p*-value
			Immediate		Aging		
			No.	%	No.	%	
N0-IDS	SA	CD	0	0.0%	0	0.0%	0.081
		AD	17	85.0%	18	90.0%	
		ADC	0	0.0%	0	0.0%	
		H	3	15.0%	0	0.0%	
		CC	0	0.0%	2	10.0%	
	F	CD	0	0.0%	1	5.0%	0.285
		AD	13	65.0%	9	45.0%	
		ADC	0	0.0%	0	0.0%	
		H	7	35.0%	8	40.0%	
		CC	0	0.0%	2	10.0%	
IDS	SA	CD	0	0.0%	2	10.0%	0.212
		AD	13	65.0%	11	55.0%	
		ADC	0	0.0%	0	0.0%	
		H	7	35.0%	5	25.0%	
		CC	0	0.0%	2	10.0%	
	F	CD	0	0.0%	6	30.0%	0.031*
		AD	12	60.0%	7	35.0%	
		ADC	1	5.0%	0	0.0%	
		H	7	35.0%	5	25.0%	
		CC	0	0.0%	2	10.0%	
p-value			0.415		0.007*		

Using: Chi-square test

*CD *Cohesive failure in dentin,* AD *Failure at interface dentin/cement,* ADC *Failure at interface cement/indirect composite,* H *Hybrid failure at both interfaces,* CC *Cohesive failure in composite

## Discussion

This study evaluated the effects of dentin sealing protocols, luting materials, and long-term water aging on the µTBS of CAD/CAM resin composite discs to dentin. Based on the statistical analysis, the first two null hypotheses were rejected, whereas the third was partially accepted. First, the dentin sealing technique significantly influenced the microtensile bond strength (*p* = 0.024), with IDS demonstrating higher overall values compared to No-IDS groups. Second, the type of luting material played a highly significant role (*p* = 0.001), as the submicron hybrid flowable composite yielded significantly higher bond strength than the self-adhesive resin cement. Third, the main effect of the aging period was not statistically significant (*p* = 0.428). However, there was a significant interaction between aging and the luting material (*p* = 0.022). This indicates that while aging did not uniformly decrease bond strength across all specimens, its effect was heavily dependent on the specific material used, confirming that immediate testing alone is insufficient to predict long-term bond stability.

### Impact of dentin sealing techniques

In the present study, IDS resulted in significantly higher mean µTBS compared to groups without IDS, particularly when combined with a flowable resin composite as the luting material. As shown in Table 2, this specific combination yielded the highest bond strength values after aging, outperforming all other experimental groups. In contrast, groups without IDS demonstrated lower overall µTBS, with the absolute lowest values observed when paired with self-adhesive resin cement after artificial aging.

The superior bond strength obtained from IDS in this study can be attributed to the immediate sealing of freshly cut dentin, which secures resin infiltration, prevents contamination, and stabilizes the hybrid layer against collagen degradation [6, 8, 32]. This durability is further enhanced by the 10-MDP monomer in the universal adhesive, which forms stable ionic bonds (MDP-Ca salts) with hydroxyapatite [33]. This chemical interaction creates a degradation-resistant nanolayer, significantly reinforcing the interface against long-term aging [34, 35].

These findings are consistent with previously published literature by Ramos et al. [6] 2025 and Magne et al. [32] 2005, who demonstrated significantly higher µTBS values in groups treated with IDS. Similar results were reported by Breemer et al. [11] 2019, Santana et al., [36] 2016 and Abo-Alazm and Safy [37] 2022, who found that IDS consistently led to improved bonding durability and optimized the adhesive interface for both composite and ceramic restorations. There is an agreement that IDS, especially when combined with universal adhesives, offers predictable outcome compared to delayed or self-adhesive approaches [38].

However, the outcome of the present study disagrees with Dalby et al. [39] 2012 who reported that IDS did not result in statistically significant improvements in shear bond strength (SBS) compared to DDS when tested using with self-etch resin cements. Additionally, Ramos et al. [6] 2025 demonstrated no difference in adhesive luting efficiency between IDS and DDS when the adhesive was separately light-cured before cementation. These disagreements may be due to the different types of resin cements and adhesives used.

### Influence of luting material type

In the present study, the type of luting material significantly influenced microtensile bond strength, with the multi-step approach utilizing a universal adhesive and flowable resin composite consistently yielding higher values than the self-adhesive resin cement. This difference was most pronounced following long-term water aging; whereas the flowable composite maintained high bond strength, the self-adhesive group demonstrated a notable reduction in bond values.

The higher bond strength of the flowable resin composite observed in this study aligns with the “resin coating” concept described by Nikaido et al. [7] 2015, where the low elastic modulus of the flowable resin acts as a stress-absorbing layer that protects the adhesive interface. This intimate adaptation to the dentin microtopography allows for uniform stress distribution during polymerization and functional loading [5]. Furthermore, as noted by Hassanien and Tolba [2] 2024, the specific submicron hybrid flowable composite used in this investigation offers a distinct advantage over traditional cements: its high filler loading combined with low viscosity creates a material that is mechanically resilient yet fluid enough to wet the dentin surface thoroughly. This dense filler network reduces water sorption and solubility, directly countering the hydrolytic degradation that typically compromises the long-term durability of adhesive interfaces [2].

Conversely, the significantly lower bond strength of the self-adhesive cement suggests that its simplified application protocol compromises interfacial integrity. As demonstrated by Monticelli et al. [40] 2008 and Goracci et al. [41] 2006, the primary limitation of self-adhesive cements is their limited capacity to demineralize dentin. The absence of a separate etching and priming step may limit the acidic monomers from adequately dissolving the smear layer or infiltrating the underlying collagen network, resulting in a superficial interaction rather than true hybridization. This drawback is exacerbated by the high viscosity of dual-cured cements, which restricts their wetting ability compared to the flowable alternative. Recent findings by Takamizawa et al. [42] 2025 further clarify this failure mode, reporting that without a separate universal adhesive application, self-adhesive cements struggle to maintain bond fatigue strength under stress. Their study confirms that the “self-adhesion” mechanism alone is often insufficient for long-term dentin bonding, necessitating the use of an auxiliary adhesive step to enhance micromechanical interlocking—a recommendation also supported by the manufacturer of the Solocem cement (Coltene, Switzerland) used in this study.

While laboratory data consistently favors multi-step protocols due to their superior chemical and micromechanical bonding, a recent systematic review of randomized clinical trials by Alvarenga et al. [43] 2024 reported no statistically significant difference in the failure rates of restorations luted with self-adhesive versus conventional cements over five years. This dissimilarity suggests that in vivo, factors such as macro-mechanical retention form and reduced operator sensitivity may compensate for the lower intrinsic bond strength of self-adhesive cements, highlighting a clear trade-off between maximum bond strength and clinical simplicity.

### Effect of aging

According to the Academy of Dental Materials guidelines on in vitro testing of dental composite bonding effectiveness to dentin and enamel using the µTBS method, aqueous storage of microspecimens for six months or longer -such as in the present study- is classified as long-term aging [29]. In the present study, under immediate conditions (48 h), no statistically significant differences were observed among the tested experimental groups. However, a statistically significant difference among the tested groups after long-term aging (6 months). Additionally, following long-term aging, the IDS group luted with flowable resin composite not only resisted degradation but exhibited a statistically significant increase in µTBS. This highlights the crucial role of long-term aging in revealing the true stability of the adhesive interface over time.

Our findings imply that the bond strength is relatively stable over time, and the tested luting materials and dentin sealing techniques are relatively resistant to degradation. The significant increase in µTBS after long-term aging in the IDS-F group may be attributed to progressive polymerization maturation and stress relaxation within the adhesive interface [44]. The pre-cured IDS layer allows the hybrid layer to mature stress-free, while the controlled hygroscopic expansion of the flowable composite compensates for shrinkage stress, tightening the marginal adaptation over time [45, 46]. Furthermore, the use of a filler-rich, submicron hybrid flowable composite likely contributes to lower water sorption and enhanced dimensional stability, resulting in more uniform stress distribution and increased bond stability following prolonged water storage [47].

This result supports the Academy of Dental Materials (ADM) guidelines where Armstrong et al. [29] emphasize, immediate testing cannot accurately predict clinical success; instead, aging is a critical determinant of the actual quality of the bond. The results also corroborate the work of Magne et al. [8] 2005 and Alrahlah et al. [46] 2013, who confirmed that highly filled flowable composites can enhance interfacial sealing, provided they are protected by a stable, hydrolytically resistant adhesive IDS layer. Contrary to our results, previous investigations have reported that bond strength decreased over time, which was attributed to degradation of the interfacial polymer network [5, 48].

Regarding the failure mode analysis, high bond strength values do not necessarily translate into enhanced resistance to failure [49]. Following aging, most groups predominantly exhibited adhesive failures, indicating that the adhesive interface remained the most vulnerable component, likely compromised by solvent evaporation or hydrolytic degradation [5]. By contrast, cohesive failures within dentin or cement were more indicative of localized stress concentration rather than reflecting intrinsically stronger adhesion [29].

This study was limited by its in-vitro design, which cannot fully simulate the complex conditions of the oral environment such as thermal cycling, occlusal loading, and enzymatic activity. The aging protocol of six months in water storage may not reflect the full spectrum of long-term intraoral degradation. Methodologically, the use of a flat dentin surface, while standard for isolating pure µTBS, does not simulate the complex 3D cavity configuration and C-factor stresses of a natural tooth preparation. Additionally, applying a 30 N static load during cementation was utilized to standardize film thickness, but this may not accurately replicate dynamic clinical seating forces. Crucially, while the µTBS test is highly discriminative, it possesses inherent shortcomings, such as high technique sensitivity during specimen sectioning and reliance on purely static tensile forces.

Furthermore, while the 3 mm thickness of the CAD/CAM composite discs was deliberately selected to test the maximum manufacturer-indicated limit for light-curing, clinical scenarios involving thicker or highly opaque restorations would necessitate the use of dual-cured protocols due to light attenuation. Finally, only one CAD/CAM resin composite, universal adhesive, resin cement, and flowable composite formulation were evaluated, and the study did not include a direct composite control group. These factors restrict the generalization of the findings. Future studies incorporating thermal and mechanical cycling, 3D cavity preparations, direct composite controls, and a broader range of luting materials are recommended to validate these results under more clinically relevant conditions. Moreover, additive testing methodologies, such as nanoleakage analysis or marginal adaptation assessments, are necessary to fully characterize the adhesive performance.

## Conclusions

Within the limitations of this in-vitro study:

- The microtensile bond strength of CAD/CAM composite to dentin is dependent on the specific choice of both the dentin sealing technique and the luting material.
- Immediate dentin sealing combined with a submicron hybrid flowable composite demonstrated the highest bond strength after long-term aging, compared to the tested self-adhesive resin cement.
- The effect of artificial aging was highly material-dependent; therefore, long-term aging is necessary to accurately evaluate the bond durability.

## Clinical relevance

The findings of this study emphasize the importance of combining appropriate dentin sealing and luting materials to optimize the long-term adhesive durability of CAD/CAM resin materials. Immediate dentin sealing using a universal adhesive, particularly when followed by luting with a submicron hybrid flowable composite, resulted in superior bond durability compared to conventional DDS and self-adhesive resin cement approaches. Further clinical studies are recommended to prove that this protocol may provide a more stable adhesive interface and longevity, reducing the risk of debonding and microleakage in non-retentive resin composite restorations in daily practice.

## Data Availability

The datasets generated and/or analyzed during the current study are available from the corresponding author upon reasonable request.

## Abbreviations

CAD/CAM: Computer-aided design and computer-aided manufacturing
IDS: Immediate dentin sealing
DDS: Delayed dentin sealing
µTBS: Microtensile bond strength
ANOVA: Analysis of Variance
10-MDP: 10-methacryloyloxydecyl dihydrogen phosphate
MMPs: Matrix metalloproteinases
UDMA: Urethane Dimethacrylate
Bis-GMA: Bisphenol A diglycidylmethacrylate
DUDMA: Diurethane dimethacrylates
4-META: 4-methacryloxyethyl trimellitic anhydride
HEMA: 2-Hydroxymethacrylate
TEGDMA: Triethyleneglycol dimethacrylates
SiO2: Silicone dioxide
CEJ: Cementoenamel junction
SBS: Shear bond strength
ADM: The Academy of Dental Materials
AD: Adhesive failure at dentin/cement interface
CC: Cohesive failure in the adhesive luting material
CD: Cohesive failure in the dentin
H: Hybrid failure at both interfaces

## Footnotes

1 Gailani, Hassan Faez Abdullah, et al. “Effect of Two Immediate Dentin Sealing Approaches on Bond Strength of Lava™ CAD/CAM Indirect Restoration.” *Materials* 14.7 (2021): 1629.

2 Faul, Franz, et al. “G* Power 3: A flexible statistical power analysis program for the social, behavioral, and biomedical sciences.” *Behavior research methods* 39.2 (2007): 175–191.
